# Correction: Considerations in Platinum and Gold Drug Design and the Synthesis of Chloro-(2,3-Diphenyl-1,3,4-Thiadiazolium-5-Thiolato-S_exo_)Gold(I): The First Gold-Mesoionic Complex of its Kind

**DOI:** 10.1155/MBD.2000.55

**Published:** 2000

**Authors:** P. F. de Athayde Filho, B. P. Howe, J. Miller

**Affiliations:** Universidade Federal da Paraíba Departamento de Química Laboratório de Tecnologia Farmacêutica CCEN, Campus I João Pessoa Paraíba CEP 58.51-970 Brazil


					CORRECTION
The authors of the paper published in Metal-Based Drugs 3 (1996), 117 must be
as indicated below:
CONSIDERATIONS IN PLATINUM AND GOLD DRUG DESIGN
THE SYNTHESIS OF CHLORO-(2,3-DIPHENYL-1,3,4-
THIADIAZOLIU M-5-THIOLATO-Sexo)GOLD(I):
THE FIRST GOLD-MESOIONIC COMPLEX OF ITS KIND
AND
P. F. de Athayde Filho, B. P. Howe and J. Miller*
Universidade Federal da Paraiba, Departamento de Quimica,
LaboratSrio de Tecnologia Farmacutica, CCEN, Campus I,
Jo&o Pessoa, Paraiba, Brazil, CEP 58.51-970.
E-mail: brianh@vm.npd.ufpb.br and millerjo@ltf.ufpb.br

				

